# Supplementary material for: Analysis of PPARγ Signaling Activity in Psoriasis
Source: Int J Mol Sci. 2021 Aug 10;22(16):8603. doi: 10.3390/ijms22168603 (PMC8395241; doi:10.3390/ijms22168603)
Supplement: Supplementary file 1 [file ijms-22-08603-s001.zip › Supplemental materials_Analysis of PPARg signaling activity in psoriasis/Pathway models/Models images and html files/PPARG expressed in cells assotiated with psoriasis/211749.html]

hair follicle


# Organ hair follicle

|  |  |
| --- | --- |
| URN | urn:agi-ncimorgan:C0221971 |
| Total Entities | 9 |
| Connectivity | 2264 |
| Name | hair follicle |

---

|  |  |
| --- | --- |
| ChildConcepts | hair bulb |
|  | isthmus of hair follicle |
|  | lower segment of hair follicle |
|  | outer hair sheath |
|  | pilar sheath |
|  | hair papilla |
|  | infundibulum of hair follicle |
|  | outer root sheath |
|  | hair follicle cell |

---

|  |  |
| --- | --- |
| Pathway | PPARG expressed in cells assotiated with psoriasis |
|  | Neighbors of hair follicle |

---

|  |  |
| --- | --- |
| MedScan ID | 8800842 |

---

|  |  |
| --- | --- |
| Alias | hair follicule |
|  | hair follicules |
|  | hair follicle |
|  | folliculus pili |
|  | hair follicles |

---

|  |  |
| --- | --- |
| NCIm ID | C0221971 |

---
